# Effects of Interfacial Passivation on the Electrical Performance, Stability, and Contact Properties of Solution Process Based ZnO Thin Film Transistors

**DOI:** 10.3390/ma11091761

**Published:** 2018-09-18

**Authors:** Liaojun Wan, Fuchao He, Yu Qin, Zhenhua Lin, Jie Su, Jingjing Chang, Yue Hao

**Affiliations:** State Key Laboratory of Wide Band Gap Semiconductor Technology, Shaanxi Joint Key Laboratory of Graphene, School of Microelectronics, Xidian University, 2 South Taibai Road, Xi’an 710071, China; ljunwan@163.com (L.W.); fuchaohe@sina.com (F.H.); qinyu_ic@163.com (Y.Q.); sujie@xidian.edu.cn (J.S.); yhao@xidian.edu.cn (Y.H.)

**Keywords:** ZnO thin film transistors, solution process, interfacial modification layers, stability, contact potential barrier

## Abstract

This paper reports low temperature solution processed ZnO thin film transistors (TFTs), and the effects of interfacial passivation of a 4-chlorobenzoic acid (PCBA) layer on device performance. It was found that the ZnO TFTs with PCBA interfacial modification layers exhibited a higher electron mobility of 4.50 cm^2^ V^−1^ s^−1^ compared to the pristine ZnO TFTs with a charge carrier mobility of 2.70 cm^2^ V^−1^ s^−1^. Moreover, the ZnO TFTs with interfacial modification layers could significantly improve device shelf-life stability and bias stress stability compared to the pristine ZnO TFTs. Most importantly, interfacial modification layers could also decrease the contact potential barrier between the source/drain electrodes and the ZnO films when using high work-function metals such as Ag and Au. These results indicate that high performance TFTs can be obtained with a low temperature solution process with interfacial modification layers, which strongly implies further potential for their applications.

## 1. Introduction

Over the past few years, metal oxide semiconductors have attracted much attention because of their high charge carrier mobility, high optical transparency in the visible region, wide band gap, etc. [1,2,3,4,5,6]. Therefore, metal oxide thin film transistors (TFT) have wide application prospects in the next generation displays, such as transparent displays, 3-dimensional (3D) displays, and active-matrix organic light emitting diode displays (AMOLEDs) [3,4,5,6,7,8]. Among these metal oxide semiconductors, ZnO with a wide band gap (3.3–3.4 eV) has been one of the most investigated materials due to its high optical transparency and good electrical properties [9,10,11]. Most of these metal oxide semiconductors with superior performance are usually manufactured using expensive vacuum deposition methods such as radio frequency (RF) sputtering [12], atomic layer deposition (ALD) [13], chemical vapor deposition [14] etc. Therefore, the manufacturing cost of these vacuum-based deposited semiconductor materials is high. Moreover, these deposition techniques cannot be used in large-area-thin-film fabrication as well as flexible devices [15]. Fortunately, a solution process has been widely used to form metal oxide thin films due to its simplicity, low fabrication cost, and large-area processability. At the same time, the solution processed metal oxide film can exhibit amorphous states with high charge carrier mobility. This is very desirable for flexible devices since amorphous oxide is insensitive to mechanical stress [3,16]. In recent years, the solution process has been widely used to form ZnO thin films at a low temperature [16,17,18,19,20,21]. Nevertheless, the relatively low intrinsic mobility of the low temperature solution processed ZnO limits the further improvement of the ZnO based TFT device performance. Thus, various methods have been used to enhance the ZnO TFT performance and stability [9,18,20,22,23].

On the other hand, previous studies have shown that adsorbed ambient species such as oxygen (O_2_) and water on the back channels of metal oxide TFTs can affect the device stability by providing acceptor or donor states [9,20,24,25]. For example, oxygen molecules as electron acceptor states can form a depletion layer and cause a positive threshold voltage (*V_th_*) shift, while water molecules as electron donor states can form an accumulation layer and cause a negative *V_th_* shift. Hence, several methods have been employed to passivate the metal oxide back channel to minimize the *V_th_* shifts. Among them, self-assembled monolayer (SAM) passivation is simple and controllable, and compatible with flexible electronics. For example, Peng et al. used octadecyltriethoxysilane (OTES) to treat indium gallium zinc oxide (IGZO) surface to enhance the device performance and electrical stability [26].

In this study, a new SAM 4-chlorobenzoic acid compound, as the surface modification layer, was employed to passivate the low temperature solution processed ZnO TFTs and improve the device performance as well as device stability. It was found that the charge carrier mobilities and stability in the air were improved after effectively passivating the surface traps of the ZnO thin films using interfacial modification layers. Moreover, the contact potential barrier between the source/drain electrodes and ZnO could be decreased when using high work-function metals, such as Ag and Au.

## 2. Materials and Methods

### 2.1. Materials

Zinc oxide (ZnO, 99.9%) was purchased from Sigma–Aldrich (Saint Louis, MI, USA). Ammonium solution (≥28%, NH_3_ in H_2_O) was obtained from Aladdin (Hamden, CT, USA). 4-chlorobenzoic acid (PCBA, 98%) and isopropyl alcohol (IPA, 99.5%) were bought from Sigma–Aldrich. All the materials were used as received without further purification.

### 2.2. Thin Films Preparation

The ZnO precursor solution (8 mg/mL) was prepared by dissolving ZnO powder directly in the ammonium solution to form a Zn(NH_3_)_4_^2+^ complex precursor solution. To make sure the ZnO powder was dissolved completely, the solution was refrigerated for several hours. Then, 4-chlorobenzoic acid (PCBA) was dissolved in isopropyl alcohol to form a 0.10 M PCBA solution.

### 2.3. Film Formation and Device Fabrication

A heavily p-doped Si wafer acted as gate electrode and the substrate, and a 200-nm-thick SiO_2_ layer thermally grown on a Si wafer with a resistivity of 0.01–0.05 Ω·cm acted as the dielectric layer. First, the Si substrate with SiO_2_ was cleaned ultrasonically in acetone, absolute ethanol, and de-ionized water. Then, to remove the surface residues and promote the formation of metal oxide thin film, an O_2_ plasma treatment (10 min, 20 W) was carried out. After that, the ZnO precursors were spin coated at 3000 rpm for 30 s to form the ZnO thin film and annealed on a hot plate at 150–300 °C for 10 min. The same spin-coating and annealing procedure was repeated once more to obtain the desired thickness (~10 nm) for ZnO thin film. Then, the substrates with the ZnO thin films were annealed on the hot plate at 150–300 °C for 40 min in ambient air. After this, for self-assembled monolayer (SAM) interfacial modification, PCBA solution was spin coated at 3000 rpm for 30 s to form interfacial modification layers. Finally, the devices were finished by thermally evaporating 100 nm thick Al or Ag source/drain electrodes on the top of the ZnO thin films with a patterned shadow mask. The channel width (*W*) and channel length (*L*) of TFTs are 1000 µm and 100 µm, respectively. The thin film transistors were determined with an Agilent 1500 semiconductor parameter analyzer under ambient in the dark on a custom probe station at room temperature. The following equation device was used to extract the field-effect mobility (*μ_sat_*) from the saturation regime of the transfer curve:

(1) $$I_{D}=\mu_{sat}C_{i}\frac{W}{2L}{\left(V_{G}-V_{th}\right)}^{2}$$

where *I_D_* accounts for the drain to source current, *μ_sat_* is the field-effect mobility, *C_i_* is the capacitance per unit area of the gate dielectric (SiO_2_ = 200 nm, *C_i_* =17.3 nF·cm^−2^), *V_G_* is gate voltage, *V_th_* is threshold voltage, and *W* and *L* are channel width and length, respectively.

### 2.4. Device Characterization

A UV-visible spectrophotometer (Perkin-Elmer Lambda 950, Waltham, MA, USA) was employed to investigate the transmittance of the ZnO film deposited on the sapphire substrate. Atomic force microscopy (AFM, Bruker Dimension Icon, Bruker, Karlsruhe, Germany) measurement was carried out to study the ZnO thin film surface morphology and the roughness. X-ray photoelectron spectroscopy (XPS) experiments were taken at the Escalab 250i using monochromatic Al-Ka (1486.6 eV) as the radiation source.

## 3. Results

**Figure 1:**
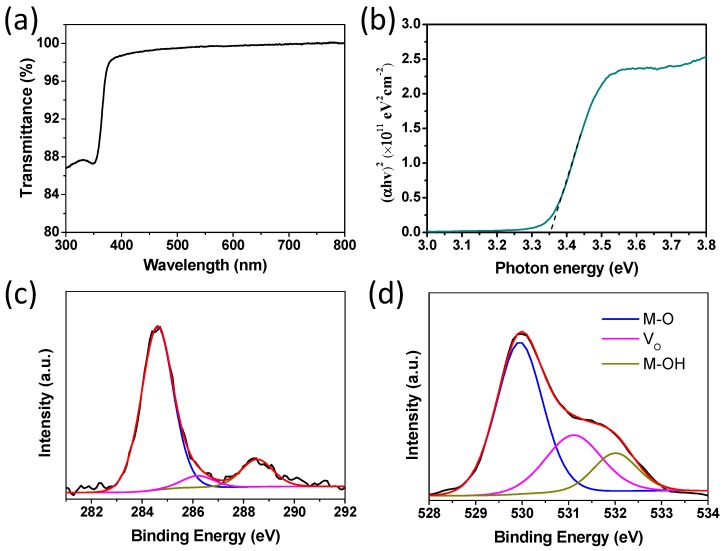
(**a**) Optical transmission spectrum of the ZnO thin film on the sapphire substrate. (**b**) The absorption coefficient as a function of photon energy of ZnO thin film annealed at 300 °C. X-ray photoelectron spectroscopy (XPS) spectra of the C1s (**c**) and O1s (**d**) core level lines for the solution processed ZnO thin film annealed at 300 °C.

ZnO thin film preparation was carried out according to a previously reported method [9,22]. Firstly, the ZnO thin film quality was evaluated by various techniques. The optical transmission spectrum of the ZnO thin film characterized by UV–Vis spectroscopy on the sapphire substrate is shown in Figure 1a. Figure 1b shows the relationship between the absorption coefficient and photon energy extracted from the transmittance spectrum. The optical bandgap of ZnO thin film was obtained by extrapolating the linear part of the plot to the X axis. The equation *T = Aexp(−αd)* was used to calculate the absorption coefficient α, where *T* is the ZnO film transmittance, *A* is a constant and approximate unity, and *d* accounts for the thickness of ZnO film. The ZnO optical bandgap can be calculated with the Tauc model from the high absorbance region: *αhυ = D(hυ − E_g_)^n^*, where *hυ* accounts for the photon energy, *E_g_* is the optical bandgap, *D* is a constant, and n is equal to 1/2. By plotting *(αhυ)^2^* versus *hυ,* the ZnO optical bandgap can be easily obtained. The ZnO thin film possesses a good transparency in the visible region and its optical bandgap is 3.35 eV, which is consistent with that of ZnO reported in the literature [20].

An XPS experiment was undertaken to investigate the chemical and structural information of the ZnO thin film in this study. Figure 1c,d display the detailed C1s (Figure 1c) and O1s (Figure 1d) scans. For C1s core levels, the reference peak used was located at 284.6 eV. The higher binding energy peaks were assigned to carbon oxide groups. The three O1s core level peaks centered at ~529.90 eV, ~531.20 eV. and ~532.10 eV corresponded to oxygen in the metal-oxide lattice (M-O), oxygen vacancies (*V_o_*). and oxygen in the hydroxide-related species (M-OH), respectively, and the ratios of these three peaks were 59.2%, 26.6% and 14.2%, respectively. More metal hydroxide and oxygen vacancies in the ZnO thin film mean more surface traps, which could affect the charge carrier mobility, leading to poor device performance of the TFT [27,28,29].

**Figure 2:**
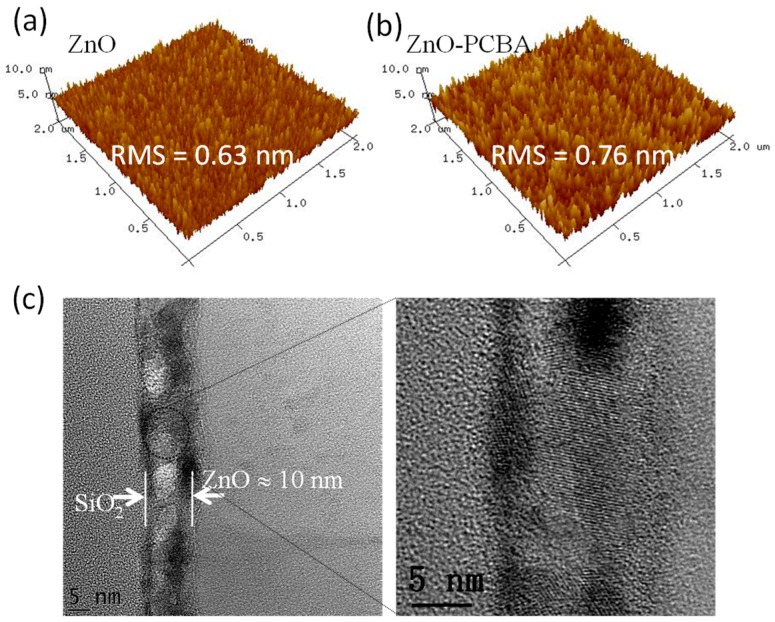
Atomic force microscopy (AFM) images of the pristine ZnO (**a**) and the ZnO with 4-chlorobenzoic acid (PCBA) interfacial modification layers (**b**). Cross-sectional high-resolution transmission electron microscopy (HR-TEM) images of the solution-processed ZnO films on the SiO_2_ substrates (**c**).

The surface morphologies of pristine ZnO and ZnO with PCBA interfacial modification layer thin films were studied by AFM, as shown in Figure 2a,b. The root mean square (RMS) surface roughnesses of the pristine ZnO and ZnO with PCBA interfacial modification layers are 0.63 nm, and 0.76 nm, respectively. The RMS values are very low and beneficial for achieving high device performance. To gain further insights into the microstructural properties of the ZnO thin film, High-resolution transmission electron microscopy (HR-TEM) was tested. Figure 2c showed the HR-TEM images from the lower to higher magnification of the ZnO cross section. The polycrystalline domains with clear lattice fringes could be observed and a lattice spacing of around 0.275 nm could be deduced from these images.

**Figure 3:**
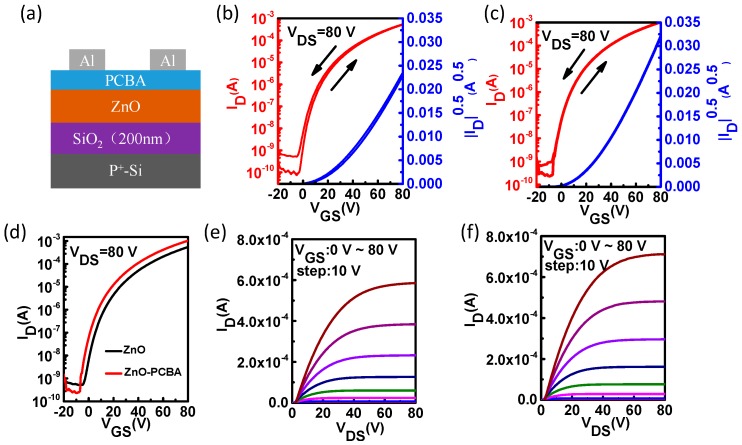
(**a**) Schematic of the thin film transistors (TFTs) structure. (**b**–**f**) Typical average transfer and output characteristics of the pristine ZnO TFTs (**b**,**e**), the ZnO TFTs with PCBA interfacial modification layers (**c**,**f**), and the transfer characteristics of the pristine ZnO TFTs and the ZnO TFTs with PCBA interfacial modification layers (**d**).

In this study, the bottom-gate top-contact TFT structure was used to evaluate the electrical properties of the ZnO thin films with and without the interfacial modification layers, as shown in Figure 3. The channel width (*W*) and channel length (*L*) of the TFT were 1000 µm and 100 µm, respectively. The transfer and output characteristics of the pristine ZnO TFTs and the ZnO TFTs with PCBA interfacial modification layers are shown in Figure 3, and the electrical characteristic parameters are summarized in Table 1. N-type response could be observed from typical output and transfer characteristics of the corresponding devices. From Figure 3 and Table 1, it was found that the device performance was improved through interfacial modification, and the ZnO TFTs with PCBA interfacial modification exhibited higher field effect mobility (4.50 cm^2^ V^−1^ s^−1^) compared to the pristine ZnO TFTs (2.70 cm^2^ V^−1^ s^−1^). At lower annealing temperature (150 °C), the devices showed a similar trend (Figures S1 and S2). The extracted field-effect mobility could be overestimated due to the geometry of the transistors and the occurring fringing current caused by the unpatterned gate electrode. From Figure 3b,c, less hysteresis of the transfer curves was observed due to fewer surface traps existing in the ZnO thin films. The trap concentration can be estimated by the displacement of *V_th_* (*N_tr_ = C_i_△V_th_/e*, where *N_tr_* is the trap concentration, *C_i_* is the gate capacitance per unit area, and *e* is the elementary charge). The surface trap concentrations of the ZnO thin film and the ZnO thin film with PCBA interfacial modification layers were calculated to be 3.7 × 10^11^ cm^−2^ and 5.5 × 10^10^ cm^−2^, respectively. It was found that through interfacial modification, the surface traps could be reduced effectively. The contact resistance (*R_C_*, between the source/drain electrodes and the semiconductor) of TFTs can be estimated using the ideal formula in the linear region of the TFT characteristics: *R_C_ = V_DS_/I_D_*. The contact resistances of the pristine ZnO TFTs and the ZnO TFTs with PCBA interfacial modification layers were calculated to be 3.50 × 10^5^ Ω and 1.34 × 10^5^ Ω, respectively. Through the interfacial modification, the contact resistance of the TFTs could be reduced effectively. Thus, we arrived at the conclusion that through interfacial modification, the device performance was improved with reduced surface traps and contact resistance. Meanwhile, *V_th_* was also reduced, indicating that the surface traps were effectively passivated and the contact resistance decreased.

**Figure 4:**
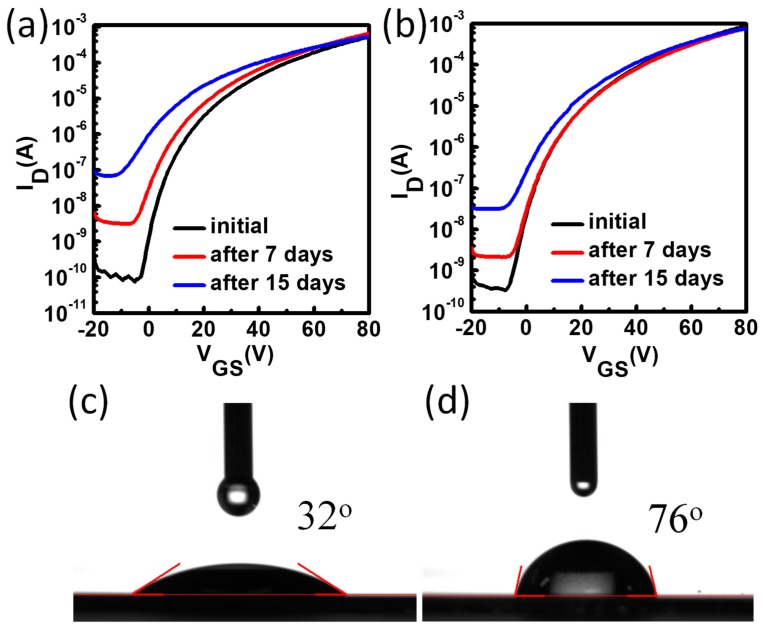
The transfer characteristics of the pristine ZnO TFTs (**a**) and the ZnO TFTs with PCBA interfacial modification layers (**b**). The water contact angle of pristine ZnO (**c**) and ZnO film with PCBA treatment (**d**).

The stability performance of the metal oxide TFTs could be evaluated by measurements of device shelf-life stability and bias stress stability. For the metal oxide TFTs, because pristine ZnO thin film can be easily aged/doped in ambient air by water molecules and oxygen, which is harmful for the device on/off ratio, the device shelf-life stability under air ambient condition plays an important role. We exposed the TFTs to ambient air with 40% relative humidity (RH) to test the device shelf-life stability, and the results are shown in Figure 4. It was found that the ZnO TFTs were unstable after 15 days because the off current increased from 10^−10^ A to 10^−7^ A and *V_th_* shift was obvious. The off current of the ZnO TFTs with PCBA interfacial modification layers also slightly increased with less *V_th_* shift after 15 days. Hence, the device shelf-life stability was improved with interfacial modification layers.

In order to obtain information for the enhanced air stability, water contact angle measurements were performed to afford the surface energy information for pristine ZnO and PCBA treated ZnO. As shown in Table 1 and Figure 4c,d, the pristine ZnO film exhibited a water contact angle of 32°, and after PCBA treatment, the contact angle increased to 76°. This indicates that the surface energy of ZnO is lowered by PCBA treatment according to Young’s equation [30]. Generally speaking, some molecules are easily absorbed onto the ZnO surface, and affect the device stability due to the absorption–desorption effect. After surface treatment, the surface becomes hydrophobic and reduces the absorption–desorption effect on the ZnO surface, which is beneficial for good air stability. This can be easily understood as PCBA has a carboxyl group in the chemical structure, and can react with the hydroxyl group at the ZnO surface to form a chemical bond, and hence the treatment reduced the hydroxyl group and surface absorbed species.

**Figure 5:**
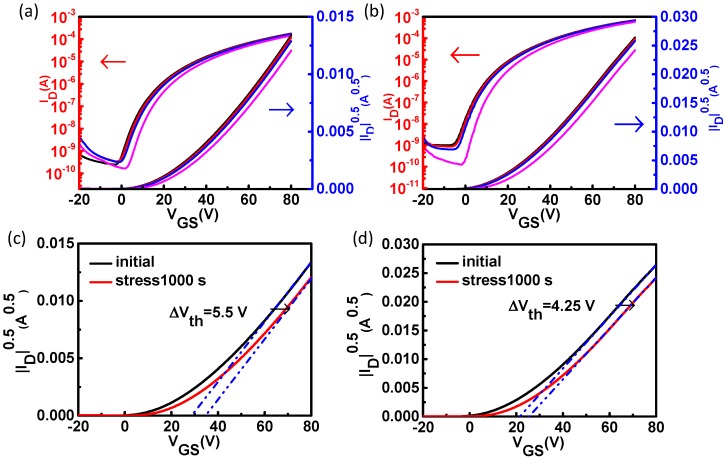
The bias stress stability of the pristine ZnO TFTs (**a**) and the ZnO TFTs with PCBA interfacial modification layers (**b**) with a gate bias of +20 V. (Black: initial; Red: stress10 s; Blue: stress100 s; Magenta: stress1000 s). The bias stress stability of the pristine ZnO TFTs (**c**) and the ZnO TFTs with PCBA interfacial modification layers (**d**) with a gate bias of +20 V for 1000 s.

The device bias stress stability which is commonly measured by the *V_th_* shift is also significant for metal oxide TFTs. Figure 5 shows the bias stress test of the pristine ZnO TFTs and the ZnO TFTs with PCBA interfacial modification layers with a +20 V gate bias. It was found that the *V_th_* shift increased with the increase of stress time. It was simply shifted along the *V_G_* axis without the transfer curve itself changing. From Figure 5, it can be seen that under constant stress condition of a gate bias of +20 V for 1000 s, the pristine ZnO TFTs and the ZnO TFTs with PCBA interfacial modification layers exhibited a *V_th_* shift of 5.50 V, and 4.25 V, respectively. Since the interfacial modification layers could passivate the ZnO surface traps, the absorbed water and oxygen induced stress instabilities could be relieved. Hence, interfacial modification layers could enhance the bias stress stability of devices.

**Figure 6:**
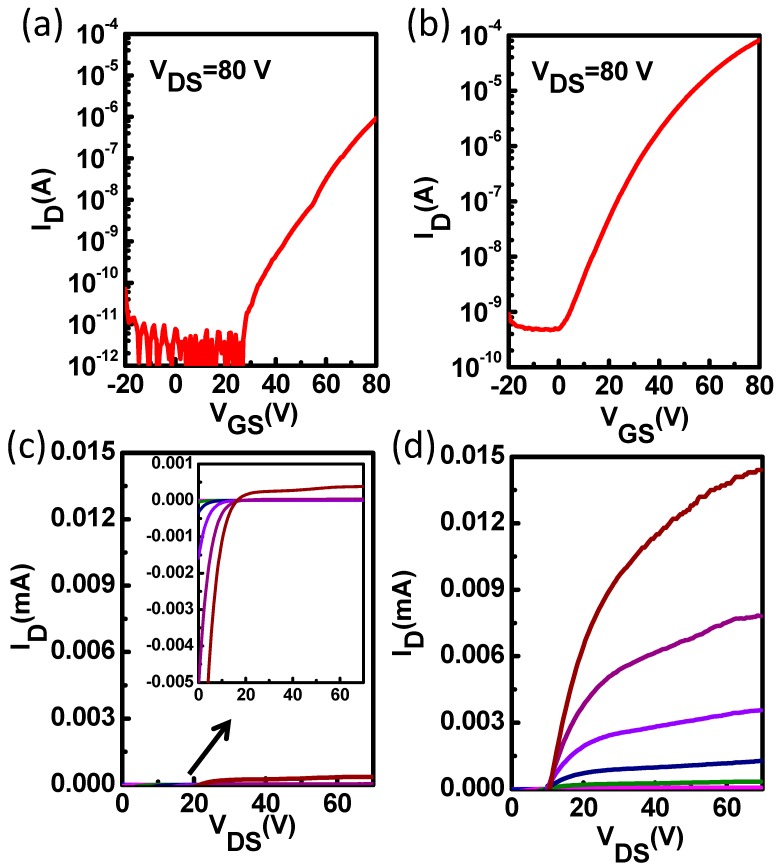
Transfer and output characteristics of the pristine ZnO TFTs (**a**,**c**) and the ZnO TFTs with PCBA interfacial modification layers (**b**,**d**) with Ag electrode.

In order to further investigate whether interfacial modification layers can optimize the interface contact between the source/drain electrodes and ZnO, we fabricated ZnO TFTs using high work-function Ag electrodes which were deposited on top of the ZnO thin film and interfacial modification layer with a shadow mask. As reported previously, Ag is a promising candidate for solution processed S/D contacts since Ag is resistant to oxidation, highly electrically conductive, and commercially available as an ink for different printing methods like inkjet printing [31,32,33,34]. Moreover, nanoparticle Ag inks could enable low temperature production of conductive films [31]. However, high contact resistance exists at the Ag electrode/semiconductor interface due to the spatial potential barrier existing at the Ag/metal oxide interface, which limits charge carrier mobilities and reduces the device performance [35]. Hence, proper interface modification could solve this problem. The average transfer and output characteristics of the pristine ZnO TFTs and the ZnO TFTs with PCBA interfacial modification layers are shown in Figure 6. The pristine ZnO TFTs exhibited a poor electron injection behavior with a low current of 10^−6^ A and large on voltage of near 30 V. The poor electrical behavior of the Ag based device is probably caused by the AgO_x_ formed at the Ag/ZnO interface, and the induced large potential barrier for electron injection [35]. However, the ZnO TFTs with PCBA interfacial modification layers exhibited an improved charge carrier mobility of 0.65 cm^2^ V^−1^ s^−1^ compared to pristine ZnO TFTs (0.025 cm^2^ V^−1^ s^−1^). Meanwhile, the drain current was significantly enhanced, and the on voltage decreased to around 0 V. The electrical characteristic parameters of pristine ZnO TFTs and ZnO TFTs with PCBA modification are summarized in Table 2. From Figure 6 and Table 2, we can also obtain the fact that through interfacial modification, the device performance was improved with reduced contact resistance and enhanced electron injection from Ag electrodes to ZnO films.

## 4. Conclusions

To summarize, we successfully fabricated solution processed ZnO thin film transistors and improved the device performance with interfacial modification layers. It was found that the ZnO TFTs with PCBA interfacial modification layers could improve the device electron mobility compared to the pristine ZnO TFTs. Furthermore, compared to the pristine ZnO TFTs, the ZnO TFTs with interfacial modification layers could improve both device bias stress stability, and shelf-life stability. Most importantly, interfacial modification layers could decrease the contact potential barrier between the source/drain electrodes and ZnO when using high work-function metals. Our results suggest that high performance TFTs can be obtained with a low temperature solution process with interfacial modification layers.

## Figures and Tables

**Table 1:** The electrical characteristics of the pristine ZnO thin film transistors (TFTs) and the ZnO TFTs with 4-chlorobenzoic acid (PCBA) interfacial modification layers with Al electrodes. The average results are calculated based on 12 devices.

Condition	Aging time	*μ_ave_* (cm^2^ V^−1^ s^−1^)	*V_th_*(V)	I_on/off_	Water Contact Angle
Pristine ZnO	initial	2.70 ± 0.21	20–25	10^6^–10^7^	32°
	after 7 days	2.80 ± 0.10	23–25	10^4^–10^5^	
	after 15 days	1.60 ± 0.30	10–14	10^3^–10^4^	
ZnO with PCBA layers	initial	4.50 ± 0.10	18–22	10^6^–10^7^	76°
	after 7 days	3.40 ± 0.10	17–20	10^5^–10^6^	
	after 15 days	2.20 ± 0.15	14–18	10^4^–10^5^	

**Table 2:** The electrical characteristics of the pristine ZnO TFTs and the ZnO TFTs with PCBA interfacial modification layers with Ag electrode.

Condition	Aging Time	*μ_ave_* (cm^2^ V^−1^ s^−1^)	*V_th_*(V)	*I_on/off_*
Pristine ZnO	initial	0.025 ± 0.03	45–60	10^5^–10^6^
	after 7 days	0.010 ± 0.04	38–45	10^3^–10^4^
ZnO with PCBA layers	initial	0.60 ± 0.05	40–48	10^5^–10^6^
	after 7 days	0.20 ± 0.02	36–42	10^4^–10^5^

## Supplementary Materials

The following are available online at http://www.mdpi.com/1996-1944/11/9/1761/s1. Figure S1: Average transfer characteristics of pristine ZnO TFTs with different annealing temperatures. The average charge carrier mobility is calculated to be around 0.65 cm^2^ V^−1^ s^−1^, Figure S2: Average transfer characteristics of ZnO TFTs without and with PCBA treatment.
