# Supplementary material for: Effects of Interfacial Passivation on the Electrical Performance, Stability, and Contact Properties of Solution Process Based ZnO Thin Film Transistors
Source: Materials (Basel). 2018 Sep 18;11(9):1761. doi: 10.3390/ma11091761 (PMC6163572; doi:10.3390/ma11091761)
Supplement: Supplementary file 1 [file materials-11-01761-s001.docx]

Supplementary information

Effects of Interfacial Passivation on the Electrical Performance, Stability, and Contact Properties of Aqueous Solution Based ZnO Thin Film Transistors

Liaojun Wan, Fuchao He, Yu Qin, Zhenhua Lin,* Jie Su, Jingjing Chang,* Yue Hao

**Figure S1.** Average transfer characteristics of pristine ZnO TFTs with different annealing temperatures. The average charge carrier mobility is calculated to be around 0.65 cm^2^ V^−1^ s^−1^.

**Figure S2.** Average transfer characteristics of ZnO TFTs without and with PCBA treatment.
